# Comparison of MiSeq, MinION, and hybrid genome sequencing for analysis of *Campylobacter jejuni*

**DOI:** 10.1038/s41598-021-84956-6

**Published:** 2021-03-11

**Authors:** Jason M. Neal-McKinney, Kun C. Liu, Christopher M. Lock, Wen-Hsin Wu, Jinxin Hu

**Affiliations:** Pacific Northwest Laboratory, US Food and Drug Administration, 22201 23rd Drive SE, Bothell, WA 98021 USA

**Keywords:** Next-generation sequencing, Microbiology

## Abstract

The sequencing, assembly, and analysis of bacterial genomes is central to tracking and characterizing foodborne pathogens. The bulk of bacterial genome sequencing at the US Food and Drug Administration is performed using short-read Illumina MiSeq technology, resulting in highly accurate but fragmented genomic sequences. The MinION sequencer from Oxford Nanopore is an evolving technology that produces long-read sequencing data with low equipment cost. The goal of this study was to compare *Campylobacter* genome assemblies generated from MiSeq and MinION data independently, as well as hybrid genome assemblies combining both data types. Two reference strains and two field isolates of *C. jejuni* were sequenced using MiSeq and MinION, and the sequence data were assembled using the software programs SPAdes and Canu, respectively. Hybrid genome assembly was performed using the program Unicycler. Comparison of the *C. jejuni* 81-176 and RM1221 genome assemblies to the PacBio reference genomes revealed that the SPAdes assemblies had the most accurate nucleotide identity, while the hybrid assemblies were the most contiguous. Assemblies generated only from MinION data using Canu were the least accurate, containing many indels and substitutions that affected downstream analyses. The hybrid sequencing approach was the most useful for detecting plasmids, large genome rearrangements, and repetitive elements such as rRNA and tRNA genes. The full genomes of both *C. jejuni* field isolates were completed and circularized using hybrid sequencing, and a plasmid was detected in one isolate. Continued development of nanopore sequencing technologies will likely enhance the accuracy of hybrid genome assemblies and enable public health laboratories to routinely generate complete circularized bacterial genome sequences.

## Introduction

The use of next-generation sequencing technologies is critical for the US Food and Drug Administration and other public health agencies to identify, characterize, and track bacterial pathogens^1,2^. These methods are especially important for foodborne pathogen outbreak investigations where there is a need to determine which isolates are identical or closely related. Genome sequencing is a rapidly evolving field with many competing technologies that produce sequence reads of differing quantity, quality, and length. The unique properties of each sequencing technology affect the way the data is assembled as well as the completeness and accuracy of the resulting genomes. In this study, we sought to examine the utility of long-read sequencing data from the Oxford Nanopore MinION to sequence the foodborne pathogen *Campylobacter jejuni*.

*Campylobacter jejuni* was chosen for analysis as this organism has a small (~ 1.7 Mb), complex genome that is AT-rich, may harbor plasmids, and contains repetitive sequences that can complicate genome assembly^3,4^. The *C. jejuni* type strains 81-176 and RM1221 examined in this study have closed reference genome sequences available, allowing us to assess the accuracy of our genome sequencing strategies. Two *C. jejuni* field isolates are also included to show the utility of our sequencing strategies in characterizing novel isolates. *Campylobacter* can be isolated from food, clinical, and environmental sources, necessitating robust genome assembly and analysis methods to track and type isolates^5^.

The bulk of bacterial genome sequencing at the US Food and Drug Administration is performed via the GenomeTrakr network, utilizing sequencing data generated by Illumina MiSeq^1^. The data produced are short paired-end reads spanning ~ 250 base pairs. The quality of the basecalls is very high, with Phred quality scores (Q-scores) often above 40^6^. In contrast, reads produced by the Oxford Nanopore MinION can be greater than 100 kbp in length, but the Q-scores are often below 10^7,8^. PacBio sequencing also produces long read data, with read accuracy that is improved by multiple sequencing passes. Completed bacterial genomes using PacBio sequencing are often considered the gold-standard for accuracy and completeness^9^. However, the PacBio instrumentation is cost-prohibitive to many laboratories while the MinION instrument costs a few thousand dollars and is highly portable. All three instruments can be used to generate bacterial genome sequences, but the accuracy and specific application of the data needs to be examined critically.

In this study, we generated de novo genome assemblies using the program SPAdes for Illumina MiSeq data, Canu for Oxford Nanopore MinION data, and UniCycler for hybrid genome assembly of both sequence data types^10–12^. The resulting assemblies were evaluated against the reference sequences for completeness and accuracy using QUAST^13^. We then determined the usefulness of the draft genome sequences for downstream analyses including whole genome comparison, gene identification and annotation, and multi-locus sequence typing. Our results demonstrate that a combination of sequencing approaches may be required to obtain the most complete and accurate genome assemblies of novel isolates.

## Results

### Sequencing of campylobacter jejuni reference strains

Two *Campylobacter jejuni* reference strains, RM1221 and 81-176, were chosen to evaluate the accuracy and completeness of genome assemblies as these strains have complete circular genomes and cultures are available from the American Type Culture Collection. Genomic DNA was extracted and sequenced using the Illumina MiSeq and Oxford Nanopore MinION platforms. The MiSeq data was generated as part of a multiplex run of 12 isolates, while the MinION sequencing was performed with a single isolate per flow cell. After basecalling with Albacore the MinION reads were down-sampled using FiltLong to estimated coverages of 40$\times$ and 200$\times$, to evaluate the amount of MinION data necessary to produce assemblies^14^. Summaries of the MiSeq data were generated using FastQC (Table 1) and summaries of the MinION data were generated using NanoStat (Table 2)^15,16^.

**Table 1:** Reference Strain MiSeq Sequencing Data.

Strain	Reads	GC%	Mean Length	Mean Q	Q30%	Mean coverage
Rm1221	928,645	33	228.9	36.37	96.22	125.04
81-176	1,788,678	32	171.7	36.39	96.49	180.66

Sequencing metrics were determined using QUAST for MiSeq data.

**Table 2:** Reference Strain MinION Sequencing Data.

Strain	Filtering	Reads	Mean Length	Mean Q	Bases	%of Reads		
						> Q7	> Q10	> Q12
Rm1221	40$\times$	3218	21,131.5	10.1	68,001,283	100	65.0	0
81-176	40$\times$	4168	16,315.2	10	68,001,798	100	47.4	0
Rm1221	200$\times$	23,874	14,241.7	9.9	340,006,757	100	36.7	0
81-176	200$\times$	28,802	10,176.8	8.9	293,111,187	100	11.4	0

Sequencing metrics were determined using NanoStat for MinION data.

The MiSeq data for RM1221 and 81-176 produced a large number of reads (928,645 and 1,788,678 reads per sample) with a mean length of 228.9 and 171.7 base pairs, respectively. The base call accuracy was high with a mean Q-score above 35 and Q30% above 95% for both reference isolates, indicating that less than 1 in 1000 bases were called incorrectly. The resulting mean coverage ranged from 125 to 180$\times$. In contrast, the filtered MinION data for Rm1221 and 81-176 had fewer reads (3218 and 4168 reads for 40$\times$ filtering) with mean lengths of 21,131.5 and 16,315.2 base pairs, respectively. The basecall accuracy was also lower, with a mean Q-score of approximately 10, indicating that approximately 1 in 10 bases were called incorrectly. The percentage of reads with Q-score > 10 was higher for 40$\times$ filtering (65% and 47.4%) than for 200$\times$ filtering (36.7% and 11.4%) due to the exclusion of lower quality sequences. The MinION run for strain 81-176 yielded only ~ 172$\times$ read coverage (293 Mbp) and did not reach the 200$\times$ cutoff (340 Mbp).

### Genome assembly of C. jejuni reference isolates

**Figure 1:**
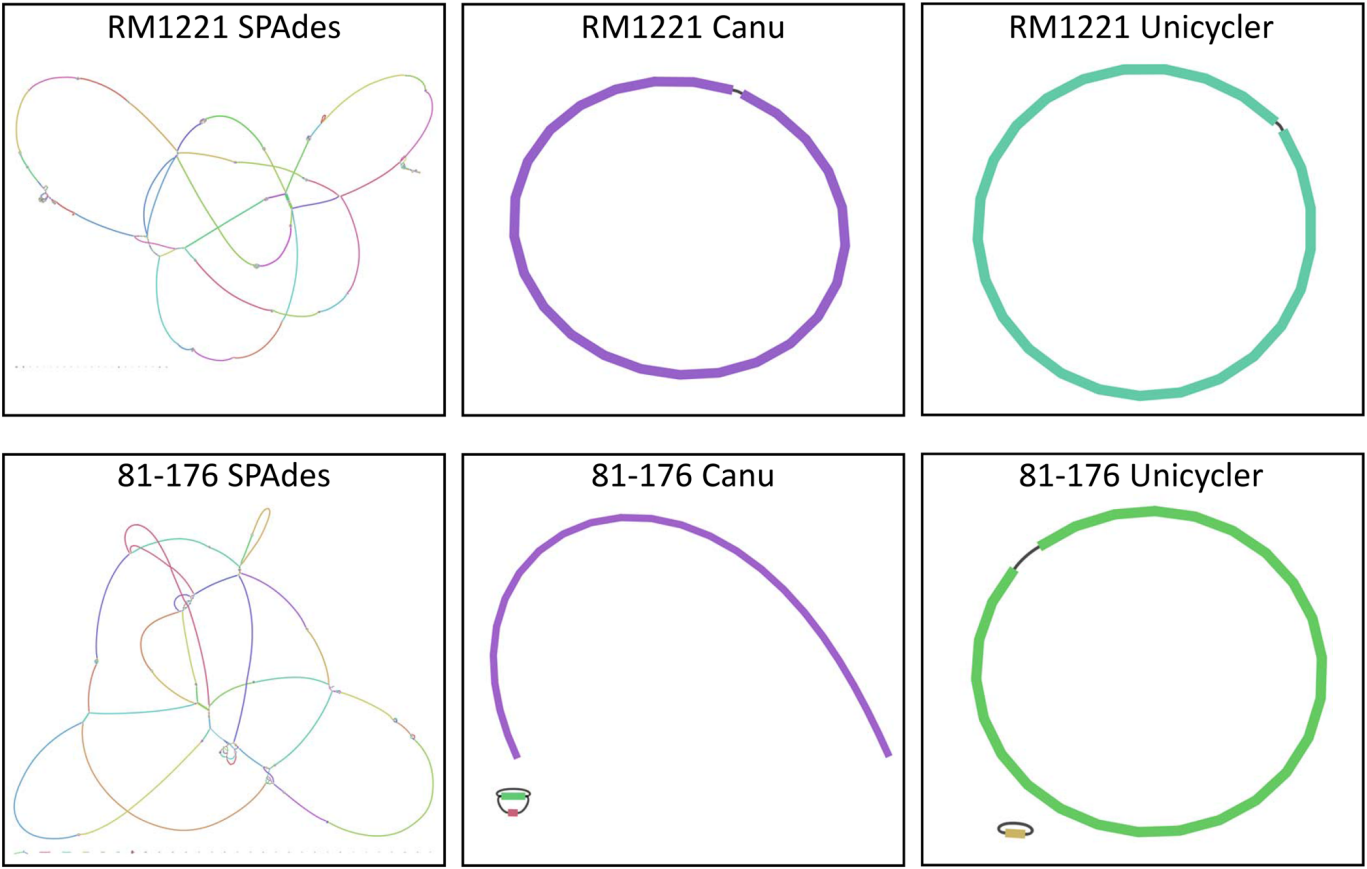
*C. jejuni* Reference Strain Assembly Graphs. The SPAdes, Canu, and Unicycler genome assembly graphs for *C. jejuni* reference strains RM1221 and 81-176 were visualized using Bandage. The colors denote different nodes, and the black connecting lines represent known overlaps. Branching node intersections are caused by multiple potential overlapping nodes.

Genomes for RM1221 and 81-176 were assembled using SPAdes for MiSeq data, Canu for MinION data, and Unicycler for hybrid assembly using both types of reads. All MiSeq reads were used for SPAdes assembly, while the MinION reads were down-sampled to an estimated 50$\times$ coverage for Canu assemblies. Assembling more than 50$\times$ coverage in Canu resulted in the program stalling and being unable to complete; however, 50$\times$ coverage is in the recommended range for bacterial genome assembly^11^. Unicycler assemblies were performed using all available MiSeq data and estimated 40$\times$ or 200$\times$ coverage for MinION read data. QUAST was used to compare the genome assemblies for RM1221 (Table 3) and 81-176 (Table 4) to the reference genomes available from NCBI (NC_003912.7 and NC_008787.1) and calculate basic assembly information. Bandage was used to visualize the assembly graphs and detect circular contigs (Fig. 1).

**Table 3:** *C. jejuni* RM1221 Assemblies.

Assembly method	Spades	Canu (50$\times$)	Unicycler (40$\times$)	Unicycler (200$\times$)
Sequence data	MiSeq	MinION	MiSeq + MinION	MiSeq + MinION
Contigs	49	1	1	1
Total length	1,754,554	1,775,387	1,775,616	1,775,624
Largest contig	196,961	1,775,387	1,775,616	1,775,624
Genome coverage	98.61	100	99.759	99.759
Total aligned length	1,754,554	1,775,383	1,773,549	1,773,557
Mismatches per 100 kbp	1.31	44.04	3.66	2.82
Circular contigs	0	1	1	1

Genomes for *C. jejuni* reference strains RM1221 were assembled using SPAdes, Canu, or Unicycler and compared to the reference genome using QUAST. The MinION data was filtered to an estimated coverage of 40$\times$, 50$\times$, or 200$\times$ using a genome size of 1.7 Mbp.

**Table 4:** *C. jejuni* 81-176 Assemblies.

Assembly method	Spades	Canu (50$\times$)	Unicycler (40$\times$)	Unicycler (200$\times$)
Sequence Data	MiSeq	MinION	MiSeq + MinION	MiSeq + MinION
Contigs	225	3	2	2
Total length	1,674,927	1,698,887	1,654,958	1,655,001
Largest contig	190,065	1,620,169	1,618,421	1,618,464
Genome coverage	98.525	99.984	100	100
Total aligned length	1,593,964	1,621,366	1,618,847	1,618,990
Mismatches per 100kbp	0.94	33.78	5.2	4.7
Circular contigs	0	1 (pVir)	2	2

Genomes for *C. jejuni* reference strains 81-176 were assembled using SPAdes, Canu, or Unicycler and compared to the reference genome using QUAST. The MinION data was filtered to an estimated coverage of 40$\times$, 50$\times$, or 200$\times$ using a genome size of 1.7 Mbp.

Assemblies with SPAdes resulted in 49 and 225 linear contigs for RM1221 and 81-176, respectively, with the longest contigs spanning 196,961 and 190,065 bp. The genome coverage for both isolates was greater than 98%, with only 1.31 and 0.94 mismatches per 100 kbp. The Bandage images for both SPAdes assemblies reveal many interconnecting nodes that could not be resolved with short-read data. No circularized contigs were detected for RM1221 or 81-176 using SPAdes assembly.

Canu produced assemblies with one circular contig of 1,775,387 bp for RM1221, and three contigs for 81-176. The largest contig for 81-176 was 1,620,169 bp, representing the bacterial chromosome. Two smaller contigs present in the assembly graph represent the pVir plasmid, although the complete circular plasmid sequence was not resolved. While the Canu assemblies were contiguous, the mismatches were greatly increased to 44.04 and 33.78 per 100 kbp.

The hybrid data assemblies performed with Unicycler were also highly contiguous, resulting in large circular contigs of 1,618,421 to 1,775,624 bp. One circular contig representing the chromosome was detected for RM1221. Two circular contigs were detected for 81-176, one for the chromosome and one for the plasmid pVir. The mismatch rate ranged from 2.82 to 5.2 mismatches per 100 kbp.

Canu assemblies performed with 50$\times$ coverage data had a greater number of errors than the Unicycler assemblies, which include high quality short reads. Increasing the amount of MinION data in the Unicycler assembly from 40 to 200$\times$ resulted in a decrease of mismatches. The RM1221 assemblies had 3.66 and 2.82 mismatches per 100 kbp and the 81-176 assemblies had 5.2 and 4.7 mismatches per 100 kbp for 40$\times$ and 200$\times$ coverage, respectively. Interestingly, Unicycler could assemble 200$\times$ MinION data with MiSeq data for all data tested, while Canu would frequently fail to complete assembly when using 200$\times$ MinION data.

### Sequencing and assembly of C. jejuni field isolates

*Campylobacter jejuni* strains R4B202 and R4B208 were selected from the archive at the US FDA Pacific Northwest Laboratory, as MiSeq sequencing was previously performed to confirm the presence of genes related to lipooligosaccharide synthesis^17^. We chose to further characterize these isolates using MinION sequencing of the same genomic DNA extracts. Summaries of the data were generated using FASTQC for MiSeq Data (Table 5) and NanoStat for MinION data (Table 6). The MinION data was down-sampled using Filtlong to an estimated coverage of 200$\times$, based on results from the reference strain assemblies. Interestingly, the MinION sequencing run for isolate R4B208 yielded only ~ 75$\times$ coverage (127.8 Mbp) and the mean read quality of 8.6 was lower than the mean read quality of 9.7 observed for isolate R4B202. We chose to utilize the limited MinION data for R4B208 to observe potential effects on assembly and analysis.

**Table 5:** Field Isolate MiSeq Data Summary.

Strain	Reads	GC%	Mean length	Mean Q	Q30%	Mean Coverage
R4B202	1,328,171	33	230.7	34.40	87.6	180.20
R4B208	5,130,797	32	163.5	36.50	97.2	494.40

Sequencing metrics for two *C. jejuni* field isolates were determined using QUAST for MiSeq data.

**Table 6:** Field Isolate MinION Data Summary.

Strain	Filtering	Reads	Mean length	Mean Q	Bases	%of Reads		
						> Q7	> Q10	> Q12
R4B202	200$\times$	15,061	22,576	9.7	340,016,499	100	23.7	0
R4B208	200$\times$	13,809	9256	8.6	127,816,314	99.7	3.2	0

Sequencing metrics for two *C. jejuni* field isolates were determined using NanoStat for MinION data.

**Figure 2:**
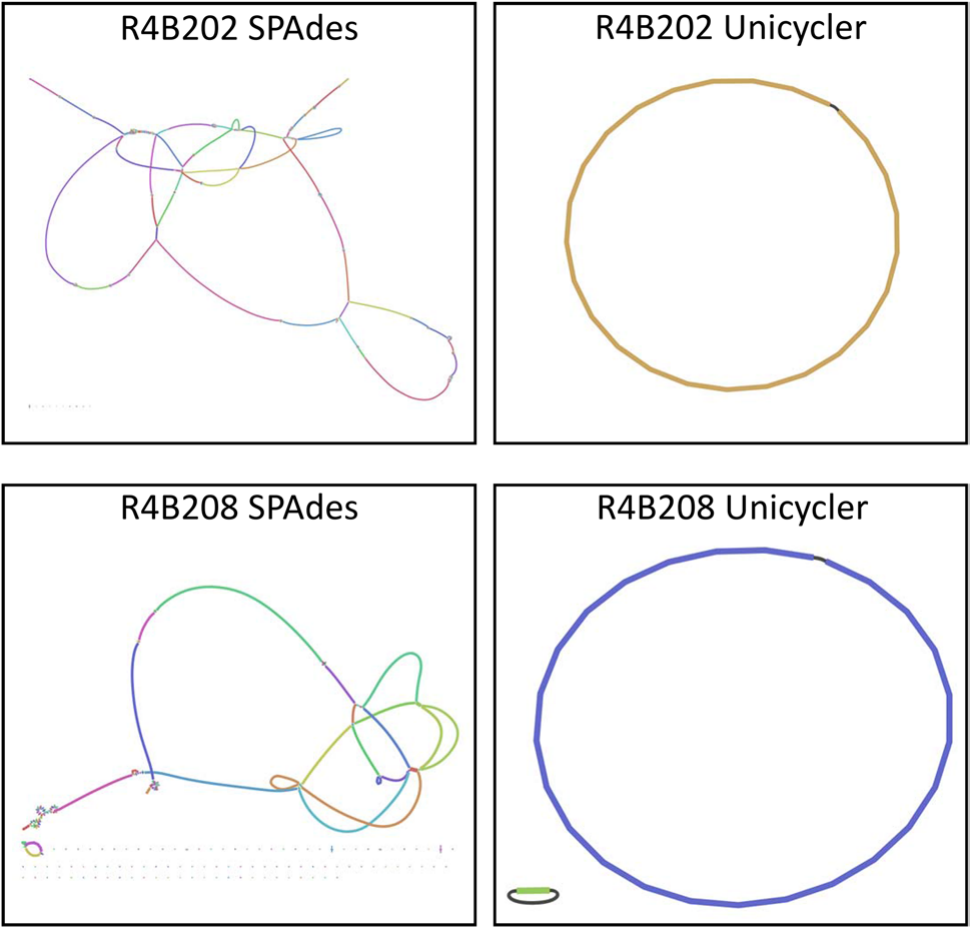
*C. jejuni* Field Isolate Assembly Graphs. The SPAdes and Unicycler genome assembly graphs for *C. jejuni* field isolates R4B202 and R4B208 were visualized using Bandage. The colors denote different nodes, and the black connecting lines represent known overlaps. Branching node intersections are caused by multiple potential overlapping nodes.

The sequencing data were assembled using SPAdes and Unicycler, and basic assembly information was calculated using QUAST without a reference genome (Table 7). SPAdes yielded 45 and 29 contigs for R4B202 and R4B208, with the largest contigs reaching 155,135 and 370,004 bp, respectively. None of the SPAdes contigs were circularized, as determined by visualization with Bandage (Fig. 2, left). In comparison, Unicycler produced one circular contig of 1,601,863 bp for the chromosome of R4B202, while R4B208 had two circular contigs; a 1,674,118 bp chromosome and a 46,407 bp plasmid (Fig. 2, right).

**Table 7:** Field Isolate Assembly Summary.

Strain	R4B202	R4B202	R4B208	R4B208
Assembly method	Spades	Unicycler	Spades	Unicycler
Contigs	45	1	29	2
Total length	1,578,385	1,601,863	1,696,813	1,720,525
Largest contig	155,135	1,601,863	370,004	1,674,118
Circular contigs	0	1	0	2

Genomes were assembled using SPAdes or Unicycler.

### Utility of MiSeq and MinION genome assemblies

**Figure 3:**
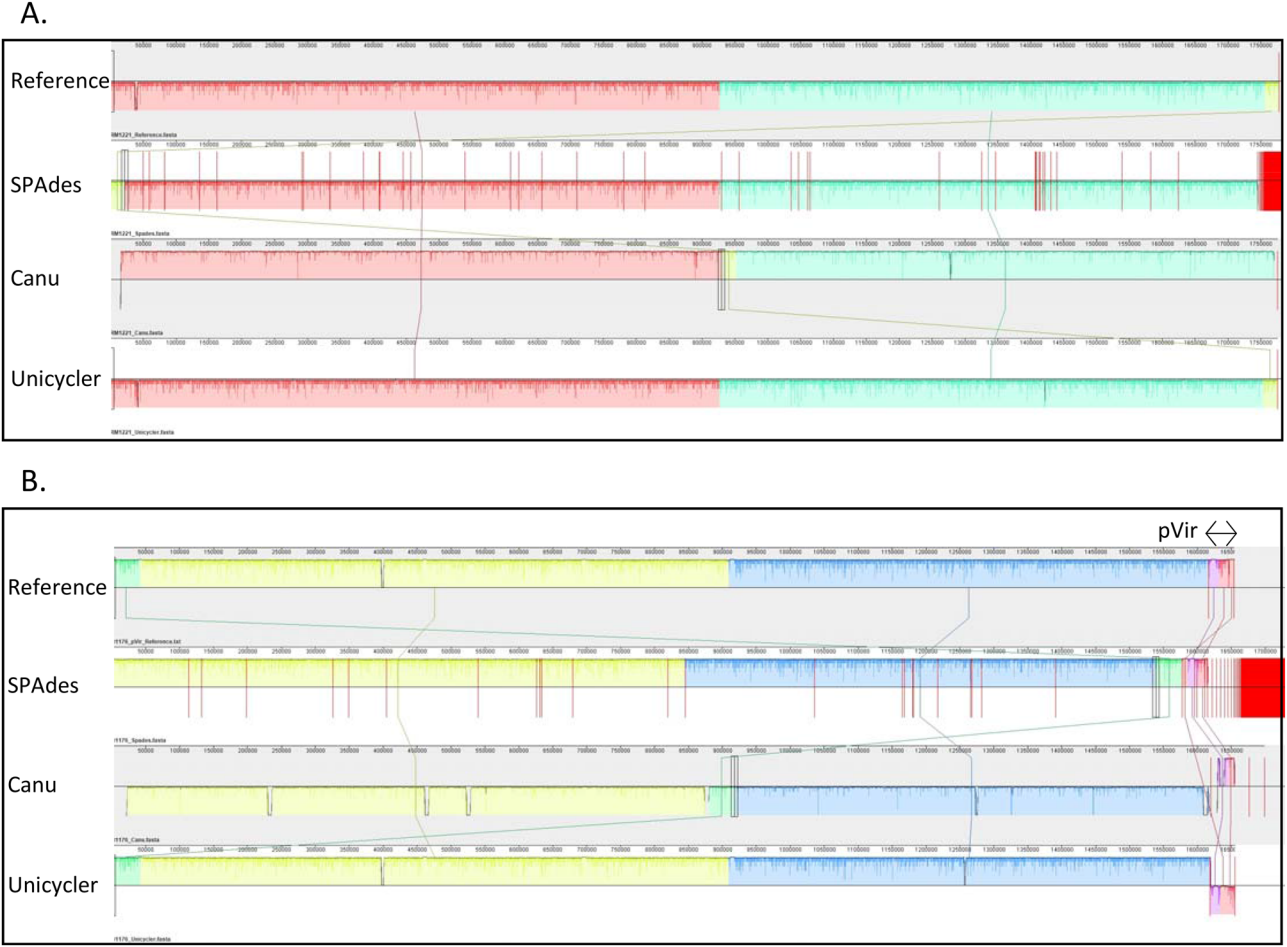
Alignment of Reference Strain Genome Assemblies. The reference genome, SPAdes assembly, Canu Assembly, and Unicycler Assembly for strains RM1221 (Panel **A**) and 81-176 (Panel **B**) were aligned using the ProgressiveMauve alignment tool. The assembly contigs were ordered according to the reference genome. The colored blocks represent homologous regions and the nucleotide identity is represented as a graph within the blocks. Vertical red lines denote contig boundaries. The *C. jejuni* 81-176 reference contig containing the pVir plasmid sequence is labeled with a double-side arrow.

**Figure 4:**
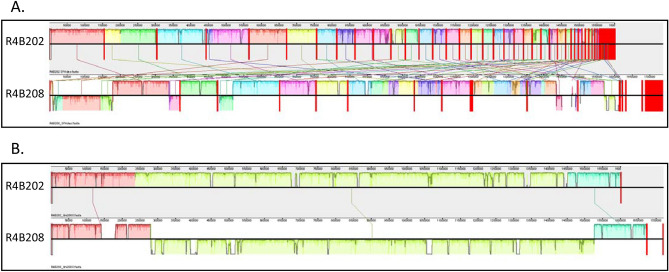
Alignment of *C. jejuni* Field Isolate Genome Assemblies. The SPAdes (Panel **A**) and Unicycler (Panel **B**) genome assemblies for *C. jejuni* isolates R4B202 and R4B208 were aligned using the ProgressiveMauve alignment tool. The SPAdes alignments were reordered 13 times in an iterative process. The colored blocks represent homologous regions and the nucleotide identity is represented as a graph within the blocks. Vertical red lines denote contig boundaries. Sequence blocks above the midline represent the forward DNA strand, while blocks below the midline represent the reverse strand to show sequence inversions.

While single nucleotide polymorphism (SNP) typing methods rely on mapping reads to a reference genome, de novo assemblies can be used to compare, type, and characterize strains without a reference. We chose to examine the utility of the genome assemblies for whole genome alignment, gene annotation, and multi-locus sequence typing (MLST). Whole genome alignments were performed using Mauve. For RM1221 and 81-176, the SPAdes assemblies were individually reordered to the reference before multiple alignment. Alignment of the assemblies for RM1221 (Fig. 3A) and 81-176 (Fig. 3B) highlight the usefulness of long-read data in direct genome comparisons, as the Canu and Unicycler assemblies do not need to be reordered. The alignments of the SPAdes and Unicycler assemblies to the reference genomes were also used to identify putative SNPs (Supplemental Table 1). For *C. jejuni* RM1221, the SPAdes assembly had 16 SNPs while the hybrid assembly had 26 SNPs. For *C. jejuni* 81-176, 3 SNPs were present in the SPAdes assembly while the hybrid assembly had 11 SNPs. The R4B202 and R4B208 SPAdes assemblies (Fig. 4A) and Unicycler (Fig. 4B) assemblies were aligned to compare the two unknown genomes. The plasmid in isolate R4B208 can be seen as a unique contig separate from the chromosome in the hybrid assembly, but is not apparent in the SPAdes assembly.

Annotation of the genome assemblies was performed using Prokka^18^. The reference genome sequences from NCBI were also annotated using Prokka to enable direct comparison of the results. A summary of the Prokka annotation results is shown in Table 8. For RM1221, the annotations for the SPAdes assembly had 1861 coding sequences (CDS) while the reference had 1871 CDS and the Unicycler assembler had 1876 CDS. For 81-176, the SPAdes assembly had 1712 CDS compared to 1674 CDS for the reference and 1672 CDS for the Unicycler assembly. The Canu assemblies for RM1221 and 81-176 yielded 4,248 and 3,938 CDS, respectively, indicating that many CDS were disrupted by errors in the Canu assemblies. Interestingly, only the Unicycler genome assembly annotations could reveal all six rRNA and 44 tRNA genes found in *C. jejuni*. Annotation of the Unicycler assembly for *C. jejuni* R4B202 yielded 1,622 CDS, six rRNA and 44 tRNA genes. Similarly, annotation of the *C. jejuni* R4B208 assembly (chromosomal and plasmid sequences) yielded 1,756 CDS, six rRNA and 44 tRNA genes. For both field isolates, the annotations of the SPAdes assemblies did not identify all rRNA and tRNA genes.

**Table 8:** Prokka annotation features.

Strain	Assembly	Contigs > 200	CDS	Genes	rRNA	tRNA
RM1221	SPAdes	77	1861	1902	2	38
RM1221	Unicycler 40$\times$	1	1876	1927	6	44
RM1221	Unicycler 200$\times$	1	1878	1929	6	44
RM1221	Canu 50$\times$	1	4248	4297	6	42
RM1221	Reference	1	1871	1922	6	44
81-176	SPAdes	195	1712	1751	2	36
81-176	Unicycler 40$\times$	2	1673	1724	6	44
81-176	Unicycler 200$\times$	2	1672	1723	6	44
81-176	Canu 50$\times$	3	3938	3985	6	40
81-176	Referenc e(pVir)	2	1674	1725	6	44
R4B202	SPAdes	55	1607	1647	2	37
R4B202	Unicycler	1	1622	1673	6	44
R4B208	SPAdes	132	1762	1801	2	36
R4B208	Unicycler	2	1756	1807	6	44

Prokka was used to annotate the genome assemblies and reference genomes to identify coding sequences (CDS) and genes. The 81-176 reference genome included both the chromosome and pVir plasmid sequence.

A direct comparison of the Unicycler assembly annotations with the reference annotations was performed for RM1221 and 81-176 to identify annotations altered by genome assembly errors (Supplemental Table 2). For RM1221, 1841 CDS were a match between the two annotations while 41 CDS did not match. For 81-176, 1668 CDS were a match between the two annotations while 7 CDS did not match. While most of the disagreements between the reference and Unicycler assemblies are caused by altered start and stop codons, there were instances where gene duplications occurred. For example, the RM1221 and 81-176 Unicycler assembly annotations lists three consecutive full-length flagellin genes, while the reference has only two consecutive flagellin genes (Supplemental Fig. 1).

MLST was performed using the *C. jejuni / C. coli* core genome MLST v1.0 typing scheme (PubMLST.org), which analyzes the sequences of 1343 loci (Table 9). Analysis of the RM1221 and 81-176 reference genomes yielded 1319 and 1322 exact allele matches, respectively. The RM1221 SPAdes assembly had 1299 exact matches and was a 98.4% match to the reference core genome sequence type (cgST). The Unicycler assembly slightly improved the results to 1318 exact matches and a 99.8% match to the reference cgST. For 81-176, the reference, SPAdes, and Unicycler assemblies all had 1322 exact matches. The UniCycler assemblies for field isolate R4B202 had 94.4% of loci match to cgST-32998 while isolate R4B208 had 94.9% of loci match to cgST-26288, indicating they may represent a novel cgST. The Canu assemblies did not provide meaningful results, as less than 50 loci were identified (not shown).

**Table 9:** Core genome MLST.

Strain	Assembly	Exact Matches	Mismatches	cgST	% Loci Match
RM1221	Reference	1319	0	1263	100.0%
RM1221	SPAdes	1299	21	1263	98.4%
RM1221	Unicycler	1318	3	1263	99.8%
81-176	Reference	1322	0	22,379	100.0%
81-176	SPAdes	1322	1	22,379	99.9%
81-176	Unicycler	1322	0	22,379	100.0%
R4B202	SPAdes	1295	85	32,998	93.7%
R4B202	Unicycler	1305	75	32,998	94.4%
R4B208	SPAdes	1302	69	26,288	94.9%
R4B208	Unicycler	1301	69	26,288	94.9%

Core genome multi-locus sequence typing was performed using the *C. jejuni/C. coli* cgMLST v1.0 typing scheme available at PubMLST.org to determine loci matches and the core genome sequence type (cgST).

## Discussion

The results of this study demonstrate the advantages and limitations of using Illumina MiSeq and Oxford Nanopore MinION sequencing to generate de novo genome assemblies of bacterial isolates. The raw data produced by the MiSeq and assembled using SPAdes was the most accurate when compared to the reference genome, but the genome assemblies were fragmented due to the short length of the reads. The fragmented nature of the genomes makes direct comparisons more difficult as the relative positions of contigs and plasmids are unknown. Most genes were properly annotated as compared to the reference, although fragmentation of the contigs disrupted some open reading frames (ORFs), resulting in a greater number of CDS. The cgMLST profiles were correct and nearly all genes in the typing scheme were represented. In contrast, the data produced by the MinION and assembled using Canu resulted in the least accurate genome assemblies. Although the genomes were contiguous and plasmids could be easily identified, the quantity of mismatches made the gene annotations and sequence typing inaccurate and not useful.

The combination of MiSeq and MinION read data to create hybrid genome assemblies with Unicycler resulted in contiguous assemblies that covered more of the reference genome than assemblies generated using MiSeq data alone. Additionally, the hybrid genome assembly was able to circularize the genomes of both strains and the pVir plasmid in 81-176. The number of nucleotide mismatches per 100 kbp in the hybrid assemblies was slightly higher than in the MiSeq assemblies (Tables 3, 4), presumably due to reliance on MinION data in regions of low MiSeq read coverage. Increasing the amount of MinION data in the assembly from 40$\times$ coverage to 200$\times$ slightly improved the accuracy of the assemblies, but both stretegies yielded complete circularized genomes. It is important to note that the number and genome size of isolates sequenced on each MinION or MiSeq run will affect the genome coverage observed, and must be optimized for the desired application. Gene annotation, cgMLST, and whole genome alignment were performed to examined the potential effects the sequence errors in the hybrid assembly may have on downstream analyses.

Gene annotation with Prokka using the hybrid genome assemblies produced the most accurate results as compared to the reference annotations (Table 4). Although the length of some ORFs were altered due to errors in the hybrid assemblies, the annotations of the Unicycler assemblies were not affected by fragmented contigs like the MiSeq assembly annotations. Importantly, the contiguity of the genomes allowed for the direct comparison of whole genomes to easily identify open reading frames that were altered (Supplemental Table 2). The hybrid genome assemblies created with Unicycler also provided better cgMLST results, as the number of exact matches increased and mismatches decreased compared to the SPAdes assemblies with MiSeq data. Taken together, these data indicate that the hybrid genome assemblies can provide a more accurate representation of gene content than MiSeq only assemblies, despite a small increase in sequence errors.

Without a reference genome, we cannot determine the absolute accuracy of the *C. jejuni* field isolate assemblies. However, based on the assembly results for the *C. jejuni* references we can assume that the draft genomes presented contain a similar amount of errors that may affect subsequent analyses. For example, the duplication of flagellin genes that we observed in the Unicycler assemblies for *C. jejuni* RM1221 and 81-176 would not have been obvious without a well-defined reference sequence. Additionally, the design of PCR primers or other sequence-specific detection strategies could be hindered by single nucleotide errors present in the genomic sequences used.

Single nucleotide polymorphism (SNP) typing is a useful tool for comparing the genetic relatedness of multiple strains. However, it requires read mapping to a universal reference sequence and the SNPs identified are limited to regions of similarity between all isolates included in the analysis. The SNPs identified and contig positioning are dependent on the reference sequence used and regions that do not align are discarded from the SNP analysis. Illumina MiSeq data is well-suited for SNP typing, as the short reads are highly accurate and can easily be mapped to a reference genome. The low base call accuracy of the long read MinION data and additional difficulty of mapping large sequences to a reference genome make it less useful for SNP typing. In contrast, the de novo genome assemblies are most useful for comparing two nearly identical isolates as the entire genome is represented and every possible nucleotide difference can be observed. In this study, more putative SNPs were observed in the Unicycler assemblies than the SPAdes assemblies when aligned to the reference genome using Mauve (Supplemental Table 1). Alignment of the complete hybrid assemblies also clearly identifies unique regions, genomic rearrangements, and plasmid contigs not visible in the fragmented SPAdes assemblies (Fig. 3).

The *C. jejuni* 81-176 reference strain used in this study has previously been reported to harbor two plasmids involved in *C. jejuni* pathogenesis and drug resistance, pVir and pTet. In this study, we detected the pVir plasmid but pTet, which confers resistance to tetracycline, was not represented in our sequences. The *C. jejuni* 81-176 isolate was obtained from the ATCC and passaged on media that did not contain tetraycline so it is possible that the plasmid was lost in the absence of selective pressure. The *C. jejuni* R4B208 field isolate possesses a 46,407 bp plasmid that was circularized and easily identified using hybrid genome assembly. Although the presence of plasmid sequences could be detected in the SPAdes assemblies, fragmentation of plasmid contigs make it more difficult to determine which contigs belong to the plasmids and which are part of the chromosome.

Two *C. jejuni* isolates with reference quality genomes were chosen for study to evaluate the accuracy of the different assemblies. However, it is possible that some sequence mismatches observed could be due to the 81-176 and RM1221 isolates used in this study varying slightly from the strains that were originally sequenced. Pascoe et al*.* demonstrated significant variation in the genomes of *C. jejuni* reference isolate NCTC 11,168 stored in different laboratories, with up to 281 SNPs observed when compared to the reference sequence^19^. In this study, the reference strains were obtained from the ATCC and passaged three times prior to DNA extraction. Additional genetic drift may have occurred prior to or during the deposition and propagation by ATCC. It is also possible that the reference genome sequences could contain some errors. It is important to note that even the SPAdes assemblies using high-quality MiSeq data had some mismatches when compared against the reference sequences.

Although hybrid genome assembly allows for closed genomic sequences, the additional costs and labor may not be worthwhile for bulk sequencing of bacterial isolates. Traditional SNP and MLST techniques using MiSeq based assemblies can accurately group isolates to identify genetic relatedness. The added costs of MinION sequencing for hybrid assemblies could be warranted when the additional detail is needed to compare highly related isolates or to characterize the complete genetic content of an isolate. During outbreak investigations it is often necessary to demonstrate that bacterial isolates from clinical samples, the environment, or food are genetically identical or closely related, especially when pursuing regulatory action. The use of complete hybrid genome assemblies would allow for the most accurate comparison between the isolates by identifying every possible nucleotide difference. In this study, more putative SNPs were identified in the de novo hybrid assemblies than in the MiSeq assemblies, and the entire chromosomal sequence was represented in the analysis.

The additional detail and contiguity of hybrid genome assembly may also be needed to fully assess the genetic content of outbreak isolates or for research purposes. For example, a previous study to characterize gene content within a lipooligosaccharide (LOS) locus in 827 *C. jejuni* genome sequences deposited via GenomeTrakr revealed that only 405 of the sequences contained the full region of interest^17^. Due to gaps in the MiSeq based assemblies between contigs the authors were unable to determine the LOS genotype of more than half the sequenced isolates. The hybrid genome assemblies produced in this study contain all the genes on a single contig without any gaps, allowing for definitive characterization of the LOS loci or any other genes of interest. The contiguity of the genomes also makes clear which portions belong to the chromosome or to plasmids. It is important to determine the complete genetic content of outbreak strains so that all potential virulence factors and drug resistance genes can be identified.

In conclusion, the addition of long-read MinION sequencing data to existing short-read MiSeq data can be used to produce accurate and complete hybrid genomes for *C. jejuni*. The assembly of MinION data alone in this study resulted in low quality genome sequences and could not be used to replace MiSeq based assembly strategies. The increased cost associated with performing two WGS techniques on bacterial isolates may be justified when characterizing novel outbreak strains or to increase the confidence in the identity of highly related isolates when pursuing regulatory actions. Future improvements to long-read sequencing and hybrid assembly methods will enable public health labs to routinely generate completed bacterial genomes for the most definitive characterization of isolates.

## Methods

### Bacterial isolates and DNA extraction

*Campylobacter jejuni* reference strains RM1221 and 81-1176 were obtained as viable stocks from the American Type Culture Collection (ATCC) and passaged three times prior to DNA extraction. The *C. jejuni* field isolates R4B202 and R4B208 were obtained from an archived collection at the US FDA Pacific Northwest Laboratory. All strains were passaged on *Campylobacter* Selective Agar (Thermo Fisher Scientific) and genomic DNA was extracted using the Qiacube with DNeasy Blood and Tissue Kit (Qiagen). Genomic DNA was quantified using a TapeStation 4200 with Genomic DNA ScreenTape (Agilent). The same genomic DNA extract was used for both MiSeq and MinION sequencing library preparation.

### DNA sequencing

Sequencing of genomic DNA extracts was performed using the Illumina Miseq and Oxford Nanopore MinION. MiSeq library preparation and barcoding was performed using the Nextera XT Library Prep Kit (Illumina) according to manufacturer’s instructions, with 12 isolates ran in multiplex per flow cell. After basecalling, paired-end reads were exported as FASTQ files for assembly. MinION library preparation was performed using the 1D^2^ library prep kit (SQK-LSK308) according to manufacturer’s instructions. One isolate was sequenced per flow cell (FLO-MIN107) for 48 h. Basecalling was performed using Albacore v2.0.1 (Oxford Nanopore), and the reads were exported as FASTQ files. The raw FASTQ files were filtered with Filtlong v0.2 to an estimated 40$\times$ or 200$\times$ coverage, based on a genome size of 1.7 Mbp^14^. MiSeq data for *C. jejuni* field isolates R4B202 and R4B208 was produced as part of a previous study^17^. All sequencing and assembly data have been deposited with the National Center for Biotechnology Information; accession information is listed in Supplemental Table 3 and 4.

### Genome assembly

SPAdes v3.12.0 was used to perform genome assembly of MiSeq data, Canu v1.8 was used to perform genome assembly of the filtered MinION data, and Unicycler v0.8.4.0 was used to perform hybrid genome assembly of MiSeq and MinION data^10–12^. Each assembly was performed using default parameters for a circular microbial genome of 1.7 Mbp. The draft genomic assemblies were uploaded to the National Center for Biotechnology Information (NCBI) Genome database (accession numbers in Supplemental Table 1).

### Analysis of genome assemblies

Basic read data and quality metrics were obtained using QUAST v5.0.2 without a reference genome for MiSeq data, while NanoStat v0.1.0 was used for MinION data^13,15^. QUAST was also used to compare the nucleotide identity of draft genome assemblies to the reference sequence. Genome assembly graphs were visualized using Bandage^20^. Reference genome assemblies for *C. jejuni* RM1221 and 81-176 chromosomal DNA, as well as the 81-176 pVir plasmid, were obtained from NCBI. Whole genome alignments were performed using Mauve with the ProgressiveMauve algorithm^21^. Rapid genome annotation was performed using Prokka, and the resulting annotations were included in sequences deposited to NCBI^18^. Genome assemblies were submitted for MLST on PubMLST.org, using the *C. jejuni*/*C. coli* core genome v1.0 typing scheme^22^. Multiple sequence alignments of the flagellin gene regions were performed using the Multalin web interface^23^.

## Supplementary Information

Supplementary Information 1.

Supplementary Information 2.

Supplementary Information 3.
