# Supplementary material for: Comparison of MiSeq, MinION, and hybrid genome sequencing for analysis of Campylobacter jejuni
Source: Sci Rep. 2021 Mar 11;11:5676. doi: 10.1038/s41598-021-84956-6 (PMC7952698; doi:10.1038/s41598-021-84956-6)

# Comparison of MiSeq, MinION, and Hybrid Genome Sequencing for Analysis of *Campylobacter jejuni*

Jason M. Neal-McKinney*, Kun C. Liu, Christopher M. Lock, Wen-Hsin Wu, and Jinxin Hu

Pacific Northwest Laboratory, US Food and Drug Administration, 22201 23rd Drive SE, Bothell, WA 98021, United States.

Correspondence:

Dr. Jason M. Neal-McKinney

jason.neal-mckinney@fda.hhs.gov

Supplemental Figure 1. Alignment of *C. jejuni* flagellin genes

DNA sequences spanning from the start of the first flagellin gene (*flaA*) to the end of the last flagellin gene (*flaB*) were aligned using the Multalin web interface. Flagellin sequences from the *C. jejuni* RM1221 (Panel A) and 81-176 (Panel B) Unicycler genome assemblies were aligned to the reference genomes. Nucleotides in red are conserved among all sequences and nucleotides in blue or black are divergent.

Supplemental Figure 1A. Alignment of *C. jejuni* RM1221 flagellin genes

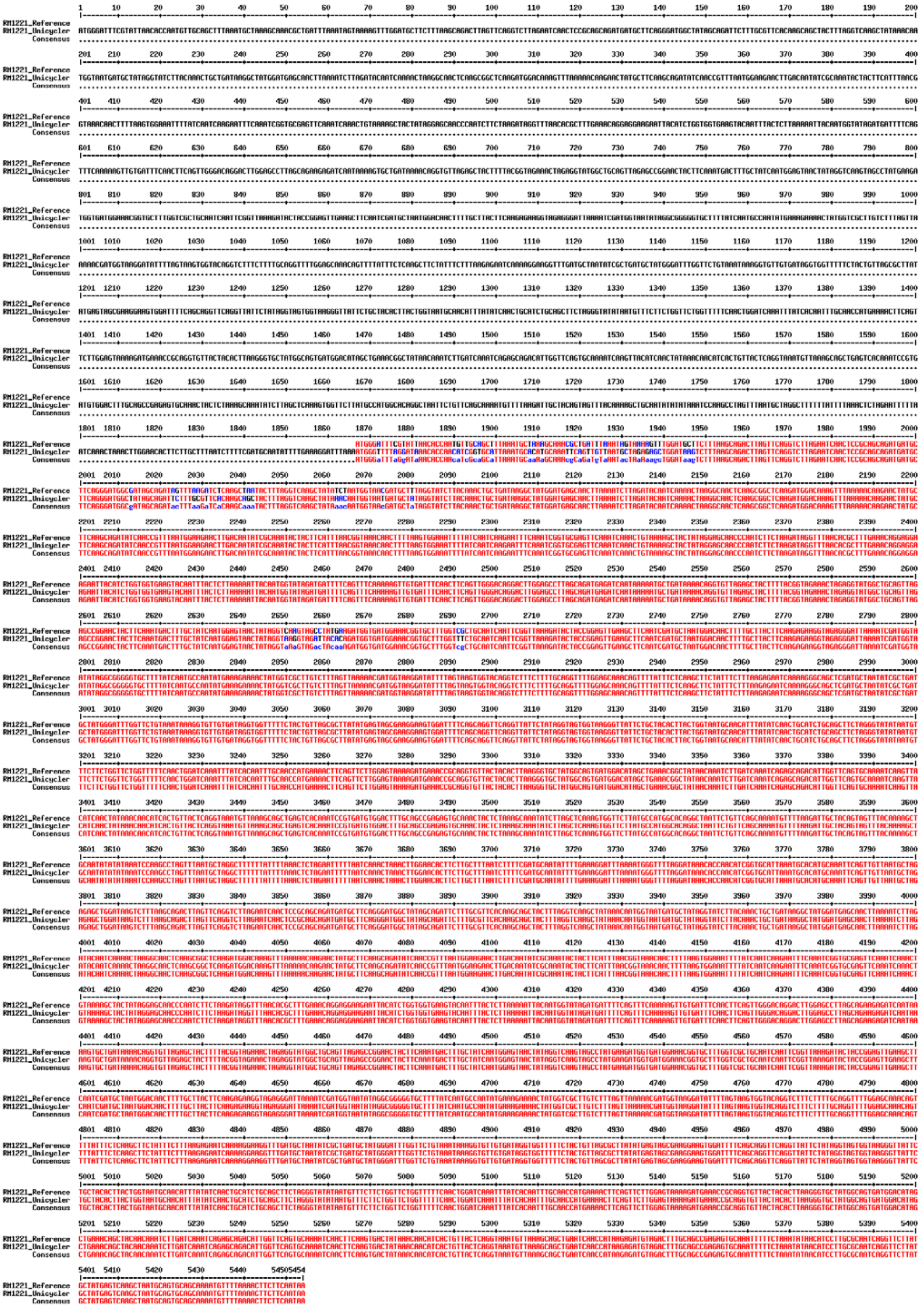

Supplemental Figure 1B. Alignment of *C. jejuni* 81-176 flagellin genes

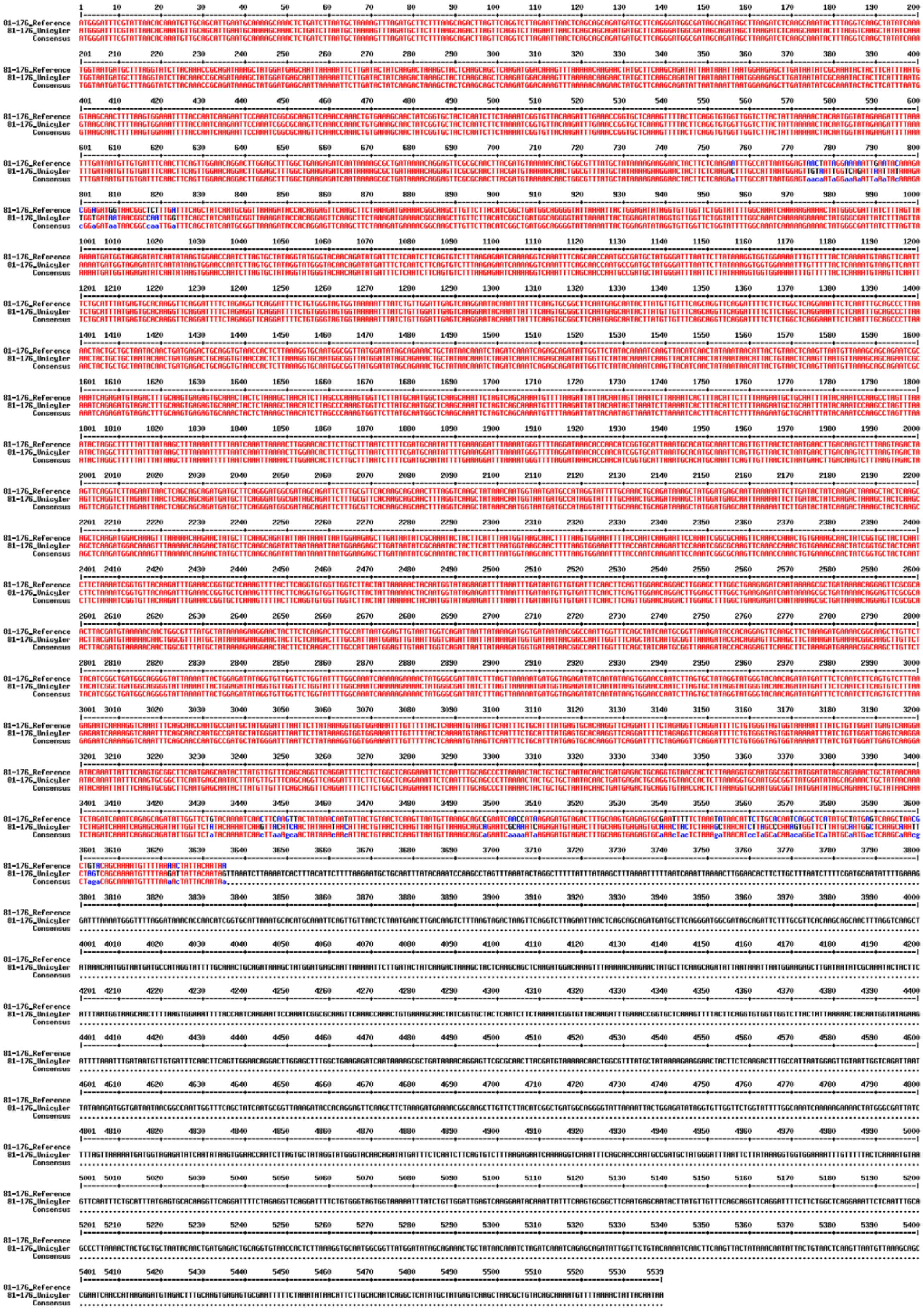

Supplement: Supplementary file 1 — Supplementary Information 1. [file 41598_2021_84956_MOESM1_ESM.pdf]
