# Supplementary material for: Comparison of MiSeq, MinION, and hybrid genome sequencing for analysis of Campylobacter jejuni
Source: Sci Rep. 2021 Mar 11;11:5676. doi: 10.1038/s41598-021-84956-6 (PMC7952698; doi:10.1038/s41598-021-84956-6)
Supplement: Supplementary file 2 — Supplementary Information 2. [file 41598_2021_84956_MOESM2_ESM.pdf]

Comparison of MiSeq, MinION, and Hybrid Genome Sequencing for Analysis of *Campylobacter jejuni*

Jason M. Neal-McKinney*, Kun C. Liu, Christopher M. Lock, Wen-Hsin Wu, and Jinxin Hu

Pacific Northwest Laboratory, US Food and Drug Administration, 22201 23rd Drive SE, Bothell, WA 98021, United States.

Correspondence:

Dr. Jason M. Neal-McKinney

jason.neal-mckinney@fda.hhs.gov

Supplemental Table 1. Reference Genome SNPs Single nucleotide polymorphisms (SNPs) were identified using Mauve to align the SPAdes and Unicycler genome assemblies to the *C. jejuni* reference genome sequences.

Reference Genome	Assembly	# of SNPs
RM1221	SPAdes	16
RM1221	Unicycler	26
81-176	SPAdes	3
81-176	Unicycler	11

Supplemental Table 2. *C. jejuni* RM1221 and 81-176 Prokka Annotation Comparison of Prokka gene annotations for the *C. jejuni* RM1221 (Table 1A) and 81-176 (Table 1B) reference sequences and hybrid genome assemblies. See Supplemental .xls file.

Supplemental Table 3. NCBI Sequence Read Archive (SRA) Accession Numbers Accession numbers for sequencing data deposited with the Nation Center for Biotechnology Information (NCBI) at ncbi.nlm.nih.gov.

Data Type	Isolate	Accession
MiSeq	RM1221	SRR12825043
MiSeq	81-176	SRR12825044
MiSeq	R4B202	SRR12825042
MiSeq	R4B208	SRR12825041
MinION 200X	RM1221	SRR12825305
MinION 200X	81-176	SRR12825307
MinION 200X	R4B202	SRR12825303
MinION 200X	R4B208	SRR12825302
MinION 40X	RM1221	SRR12825304
MinION 40X	81-176	SRR12825306
MinION 50X	RM1221	SRR13088116
MinION 50X	81-176	SRR13088115

Supplemental Table 4. NCBI Genome Accession Numbers Accession numbers for genome assemblies deposited with the Nation Center for Biotechnology Information (NCBI) at ncbi.nlm.nih.gov.

Assembler	Sequence Data	Strain	Accession
SPAdes	MiSeq	RM1221	JADWZE000000000
SPAdes	MiSeq	81-176	JADWZF000000000
SPAdes	MiSeq	R4B202	JADWZD000000000
SPAdes	MiSeq	R4B208	JADWZC000000000
Canu	MinION	RM1221	CP066243
Canu	MinION	81-176	JADWZJ000000000
Unicycler	MiSeq and MinION 40X	RM1221	CP066242
Unicycler	MiSeq and MinION 40X	81-176	JADWZI000000000
Unicycler	MiSeq and MinION 200X	RM1221	CP066241
Unicycler	MiSeq and MinION 200X	81-176	JADWZH000000000
Unicycler	MiSeq and MinION 200X	R4B202	CP063357
Unicycler	MiSeq and MinION 200X	R4B208	JADWZG000000000
